# Crystal structure of *catena*-poly[bis­(tetra­ethyl­ammonium) [tetra­aqua­tris(μ-dicyanamido-κ^2^ *N* ^1^:*N* ^5^)bis(dicyanamido-κ*N* ^1^)di­cobaltate(II)] dicyanamide]

**DOI:** 10.1107/S2056989016016637

**Published:** 2016-10-21

**Authors:** Chen Liu, Annaliese E. Thuijs, Khalil A. Abboud

**Affiliations:** a Department of Chemistry and Environmental Science, Grenfell Campus, Memorial University of Newfoundland, Corner Brook, NL, A2H 5G4, Canada; b Department of Chemistry, University of Florida, Gainesville, FL, 32611-7200, USA

**Keywords:** crystal structure, metal dicyanamide coordination polymer, cation template, tetra­ethyl­ammonium, O—H⋯N hydrogen bonding, crystal structure

## Abstract

The structure of the title compound, [N(C_2_H_5_)_4_]_2_[Co_2_(C_2_N_3_)_5_(H_2_O)_4_](C_2_N_3_), is a new example of a metal–dicyanamide coordination polymer which exhibits a unique three-dimensional framework of covalently linked Co^II^ chains. All bridging dicyanamide ligands in the title structure are in the *μ*_1,5_-bridging mode. The anionic Co^II^-dicyanamide network is templated by tetra­ethyl­ammonium cations residing in a series of channels extending along the *b* axis where additional non-coordinating dicyanamidate anions are also located. The framework structure is further stabilized by O—H⋯N hydrogen bonding between aqua ligands and dicyanamido ligands or the dicyanamide anion. In addition, C—H⋯N inter­actions are present between the tetra­ethyl­ammonium cations and dicyanamide amide nitro­gen atoms.

## Abstract 2

The title structure comprises a cation-templated anionic Co^II^-dicyanamide network composed of *μ*_1,5_-dicyanamide-bridged Co^II^ chains inter-connected *via μ*_1,5_-dicyanamide bridges.

## Chemical context   

Dicyanamide is a versatile ligand in the design and synthesis of coordination polymers due to its ability to coordinate to transition metal ions in a number of different modes involving some or all of its three nitro­gen atoms (Batten & Murray, 2003 ▸). Reactions between transition metal ions and dicyanamide have mainly produced three types of coordination polymers, including the neutral binary systems of *M* ^II^(dca)_2_ (dca = dicyanamide), complexes derived from *M* ^II^(dca)_2_ by including a co-ligand, and cation-templated anionic [*M* ^II^(dca)_*n*_]^(2–*n*)^ (*n* = 3,4) networks (Batten & Murray, 2003 ▸). These metal–dicyanamide coordination polymers exhibit a wide range of structures, from three-dimensional rutile-like structures for *M* ^II^(dca)_2_ to networks of reduced dimensions when a co-ligand or a counter-cation is included. Much of the inter­est in metal–dicyanamide coordination polymers has been focused on their structural diversities and their magnetic properties, particularly the long-range ferromagnetic ordering observed in some of the *M* ^II^(dca)_2_ networks (Kurmoo & Kepert, 1998 ▸). Compared to co-ligand-modified derivatives of *M* ^II^(dca)_2_ complexes, there are fewer examples of cation-templated anionic [*M* ^II^(dca)_*n*_]^(2–*n*)^ (*n* = 3,4) networks. We recently prepared the title compound, (N(C_2_H_5_)_4_)_2_[Co_2_(H_2_O)_4_(C_2_N_3_)_5_](C_2_N_3_), as a new example of a cation-templated metal–dicyanamide coordination polymer. The title structure presents a unique single three-dimensional network of covalently linked chains rather than a two-dimensional structure as commonly observed in many other metal–dicyanamide coordination polymers.
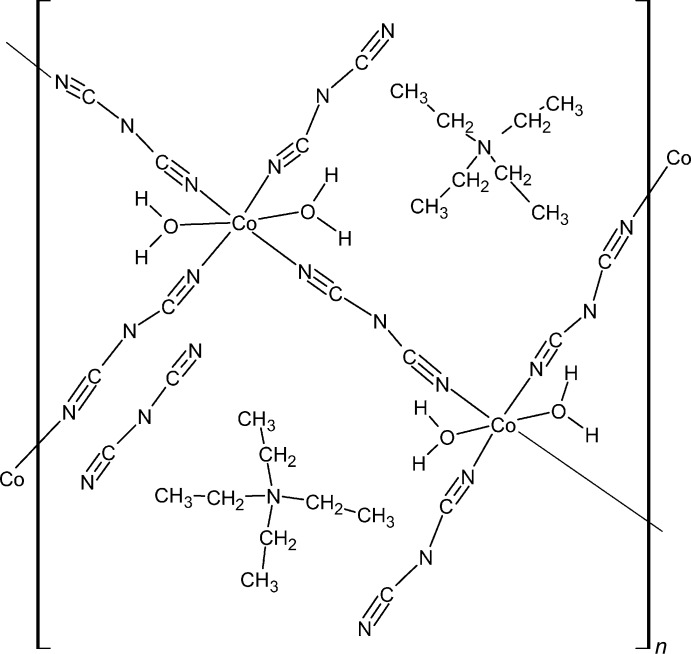


## Structural commentary   

**Figure 1:**
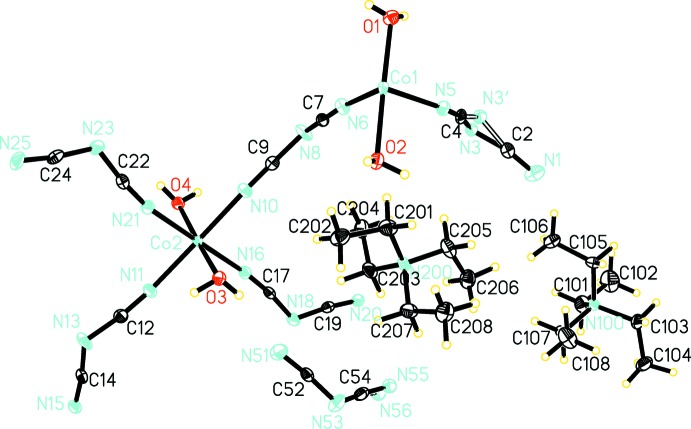
A view of the asymmetric unit of the title compound, showing the atom labeling. Displacement ellipsoids are drawn at 50% probability level. All disordered components are shown.

In the asymmetric unit of the title coordination polymer, there are two Co^II^ ions, Co1 and Co2, linked by a *μ*_1,5_-bridging dicyanamide ligand (Fig. 1 ▸). Co2 is coordinated by three dicyanamide ligands *via* their terminal nitro­gen atoms and two *trans*-positioned aqua ligands, forming an N_4_O_2_ octa­hedral coordination sphere that is slightly elongated along the two Co—O bonds. Co1 is coordinated by one dicyanamide ligand *via* its terminal nitro­gen atom and two *trans*-positioned aqua ligands. The likewise distorted octa­hedral N_4_O_2_ coordination sphere around Co1 is completed by additional bonds to N20^ii^ and N15^xi^ [symmetry codes: (ii) *x*, 2 − *y*, −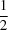 + *z*; (xi) −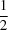 + *x*, 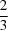 − *y*, −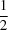 + *z*] of two symmetry-generated dicyanamide ligands. The asymmetric unit also contains two tetra­ethyl­ammonium counter-ions and a non-coordinating dicyanamide anion. Two nitro­gen atoms, N3/N3′ of one terminal ligand and N55/N56 of the anion, are disordered and were refined over two sets of sites.

**Figure 2:**
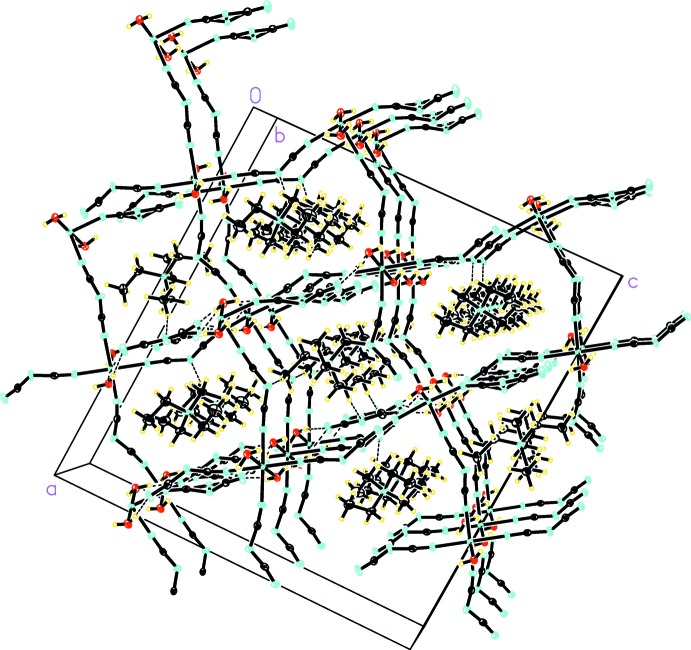
Crystal packing of the title compound, showing hydrogen bonds as dashed lines.

In the crystal, a *μ*_1,5_-dca-bridged corrugated Co^II^ chain can be seen parallel to the [101] direction and is composed of Co^II^ ions generated by a *c* glide plane parallel to the *ac* plane. Among the four dca ligands on each Co^II^ cation, three are in *μ*_1,5_-bridging mode with two bridging within the same chain and one bridging to another chain. The remaining fourth dca ligand [N1,C2,N3(N3′),C4,N5 for Co1 and N21,C22,N23,C24,N25 for Co2] is mono-dentate non-bridging. In the chain, the distances between two neighboring Co^II^ atoms linked by *μ*_1,5_-dca ligands alternate between 8.1484 (8) Å (Co1⋯Co2) and 8.5620 (8) Å [Co2⋯Co1^ix^, symmetry code: (ix) 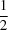 + *x*, 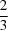 − *y*, 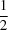 + *z*]. All of the inter-chain Co⋯Co distances across *μ*_1,5_-dca bridges are of the same length, *viz*. 8.5517 (8) Å. These distances are similar to other single *μ*_1,5_-dca bridges reported in the literature (van der Werff *et al.*, 2001 ▸; Schlueter *et al.*, 2005 ▸; Biswas *et al.*, 2006 ▸). In the title structure, each chain is linked to four other chains generated by a *c* glide plane *via* the inter-chain *μ*_1,5_-dca ligands at each Co^II^ site [Co1⋯Co2^ii^, Co2⋯Co1^xii^; symmetry code: (xii) *x*, 2 − *y*, 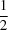 + *z*; Co1^ix^⋯Co2^v^; symmetry code: (ix) 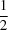 + *x*, 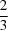 − *y*, 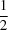 + *z*, and Co2^ix^⋯Co1^x^; symmetry code: (x) 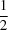 + *x*, −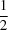 + *y*, 1 + *z*], resulting in a single three-dimensional network of covalently linked parallel chains. This is in contrast to the layered structures observed in a number of [*M* ^II^(dca)_*n*_]^(2−*n*)^ (*n* = 3, 4) networks that exhibit parallel sheets linked in the third dimension *via μ*_1,5_-dca ligands (Batten & Murray, 2003 ▸; Schlueter *et al.*, 2005 ▸; Biswas *et al.*, 2006 ▸). As a result of the mono-dentate non-bridging dca ligands in the title compound, the commonly observed (4,4) nets in other metal–dca networks are absent in its structure. However, channels extending along the *b* axis can still be seen in the title structure and these are occupied by columns of tetra­ethyl­ammonium cations (Fig. 2 ▸) and dca^−^ anions. Similar to other cation-templated anionic [*M* ^II^(dca)_*n*_]^(2−*n*)^ (*n* = 3, 4) networks, inter­penetration is not observed in the title structure due to the presence of tetra­ethyl­ammonium cations in the void space, making these structures potential candidates for investigating their ability of storing guest mol­ecules.

## Supra­molecular features   

Hydrogen bonding is generally not observed amongst the neutral *M* ^II^(dca)_2_ networks. Upon introducing co-ligands or counter-ions, the derived *M* ^II^(dca)_2_ *L_n_* (*L*: co-ligand) and cation-templated [*M* ^II^(dca)_*n*_]^(2–*n*)^ (*n* = 3,4) complexes display hydrogen bonding. In most of the *M* ^II^(dca)_2_ derivatives, the hydrogen bonds are of non-classical C—H⋯*X* (*X* = N, O) types (Tong *et al.*, 2003 ▸; Biswas *et al.*, 2006 ▸; Rajan *et al.*, 2013 ▸). In the title structure, hydrogen bonds are mainly of the classical O—H⋯N type between OH groups of coordinating water mol­ecules and nitro­gen atoms of the non-coordinating dca^−^ anion or the mono-dentate non-bridging dca ligands. Some hydrogen bonds in the title structure are bifurcated between two donor water mol­ecules located on two neighboring chains stacked along the *b* axis and thus hold these chains in place along the *b* axis. Chains related by *c* glide-plane symmetry are primarily linked *via* single *μ*_1,5_-dca ligands as described in the previous section, but are further stabilized by hydrogen bonds across the non-coordinating dca^−^ anions (N51 and N55/56) and by hydrogen bonds involving N1 and N25 of the mono-dentate non-bridging dca ligand. In addition to the O—H⋯N hydrogen bonds, C—H⋯N hydrogen bonds are also present in the title structure between C—H groups of the tetra­ethyl­ammonium cations and dicyanamide amide nitro­gen atoms (Fig. 2 ▸). The hydrogen-bond lengths and angles are summarized in Table 1 ▸.

## Synthesis and crystallization   

The title compound was prepared in a reaction where Co(NO_3_)_2_·6H_2_O (1 mmol, 291 mg), NaN(CN)_2_ (1.5 mmol, 133.55 mg), and (C_2_H_5_)_4_NCl (1.5 mmol, 249 mg) were dissolved in 40 ml of deionized water to produce a dark-red solution. Upon standing for one month, irregularly shaped red crystals (95 mg, yield 22.4%) suitable for X-ray diffraction were collected by vacuum filtration and washed with deionized water. Selected IR bands (KBr, cm^−1^): 3370 (O—H), 2977 (C—H), 2300, 2273, 2255, 2236, 2182, 2141 (C≡N), 1365 (C—N amide), 1172 (C—N amine). Elemental analysis calculated for C_28_H_48_Co_2_N_20_O_4_: C 39.72, H 5.71, N 33.08%. Found: C 39.81, H 5.37, N 32.74%.

## Refinement details   

Crystal data, data collection and structure refinement details are summarized in Table 2 ▸. C-bound H atoms were positioned geometrically (C—H = 0.98/0.99 Å) and allowed to ride with *U*_iso_(H) = 1.2/1.5*U*_eq_(C) whereby methyl H atoms were allowed to rotate around the corresponding C—C bond. Two nitro­gen atoms, N3/N3′ and N55/N56, were disordered and refined in two parts each with their respective site-occupation factors refined dependently [occupation ratios of 0.33 (4):0.67 (4) and 0.48 (3):0.52 (3), respectively] and with independent *U*_eq_ parameters for each of the N atoms. All of the water H atoms were obtained from a difference Fourier map and refined freely.

## Supplementary Material

Crystal structure: contains datablock(s) I. DOI: 10.1107/S2056989016016637/wm5330sup1.cif

Structure factors: contains datablock(s) I. DOI: 10.1107/S2056989016016637/wm5330Isup2.hkl

CCDC reference: 1510337

Additional supporting information: crystallographic information; 3D view; checkCIF report

## Figures and Tables

**Table 1:** Hydrogen-bond geometry (Å, °)

*D*—H⋯*A*	*D*—H	H⋯*A*	*D*⋯*A*	*D*—H⋯*A*
O1—H1*A*⋯N25^i^	0.79 (5)	2.08 (5)	2.862 (4)	168 (4)
O1—H1*B*⋯N55^ii^	0.71 (4)	2.09 (4)	2.792 (7)	168 (4)
O1—H1*B*⋯N56^ii^	0.71 (4)	2.22 (4)	2.922 (9)	170 (4)
O2—H2*A*⋯N25^iii^	0.84 (4)	2.03 (4)	2.862 (4)	172 (3)
O2—H2*B*⋯N55^iv^	0.71 (4)	2.27 (4)	2.963 (8)	169 (4)
O2—H2*B*⋯N56^iv^	0.71 (4)	2.12 (4)	2.819 (8)	171 (4)
O3—H3*A*⋯N51	0.77 (4)	2.07 (4)	2.822 (4)	165 (4)
O3—H3*B*⋯N1^v^	0.84 (6)	2.09 (6)	2.904 (4)	162 (5)
O4—H4*A*⋯N51^vi^	0.74 (4)	2.23 (4)	2.958 (4)	172 (4)
O4—H4*B*⋯N1^vii^	0.77 (5)	2.07 (5)	2.833 (4)	169 (5)
C105—H10*L*⋯N18^i^	0.99	2.57	3.439 (5)	146
C202—H20*E*⋯N8^viii^	0.98	2.60	3.579 (5)	173
C204—H20*H*⋯N53^vi^	0.98	2.49	3.316 (4)	142

Symmetry codes: (i) 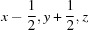; (ii) 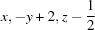; (iii) 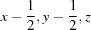; (iv) 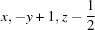; (v) 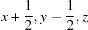; (vi) 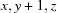; (vii) 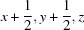; (viii) 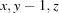.

**Table 2:** Experimental details

Crystal data	
Chemical formula	(C_8_H_20_N)_2_[Co_2_(C_2_N_3_)_5_(H_2_O)_4_](C_2_N_3_)
*M*_r_	846.72
Crystal system, space group	Monoclinic, *C* *c*
Temperature (K)	100
*a*, *b*, *c* (Å)	23.9836 (19), 7.3271 (6), 22.6809 (17)
β (°)	94.4257 (14)
*V* (Å^3^)	3973.8 (5)
*Z*	4
Radiation type	Mo *K*α
μ (mm^−1^)	0.90
Crystal size (mm)	0.16 × 0.16 × 0.10

Data collection	
Diffractometer	Bruker APEXII DUO CCD
Absorption correction	Analytical based on measured indexed crystal faces (*SHELXTL2014*; Sheldrick, 2015*a* ▸)
*T*_min_, *T*_max_	0.898, 0.947
No. of measured, independent and observed [*I* > 2σ(*I*)] reflections	26031, 9018, 8397
*R*_int_	0.026
(sin θ/λ)_max_ (Å^−1^)	0.649

Refinement	
*R*[*F* ^2^ > 2σ(*F* ^2^)], *wR*(*F* ^2^), *S*	0.028, 0.070, 1.02
No. of reflections	9018
No. of parameters	519
No. of restraints	2
H-atom treatment	H atoms treated by a mixture of independent and constrained refinement
Δρ_max_, Δρ_min_ (e Å^−3^)	1.10, −0.22
Absolute structure	Flack *x* determined using 3859 quotients [(*I* ^+^)−(*I* ^−^)]/[(*I* ^+^)+(*I* ^−^)] (Parsons *et al.*, 2013 ▸)
Absolute structure parameter	0.016 (4)

Computer programs: *APEX2* and *SAINT* (Bruker, 2008 ▸), *SHELXT2014* (Sheldrick, 2015*a* ▸), *SHELXL2014* (Sheldrick, 2015*b* ▸), *XP* in *SHELXTL-Plus* (Sheldrick, 2008 ▸) and *publCIF* (Westrip, 2010 ▸).

## supplementary crystallographic information

### Crystal data

**Table:**

(C_8_H_20_N)_2_[Co_2_(C_2_N_3_)_5_(H_2_O)_4_](C_2_N_3_)	*F*(000) = 1768
*M**_r_* = 846.72	*D*_x_ = 1.415 Mg m^−^^3^
Monoclinic, *C**c*	Mo *K*α radiation, λ = 0.71073 Å
*a* = 23.9836 (19) Å	Cell parameters from 9967 reflections
*b* = 7.3271 (6) Å	θ = 2.0–28.0°
*c* = 22.6809 (17) Å	µ = 0.90 mm^−^^1^
β = 94.4257 (14)°	*T* = 100 K
*V* = 3973.8 (5) Å^3^	Irregular, red
*Z* = 4	0.16 × 0.16 × 0.10 mm

### Data collection

**Table:**

Bruker APEXII DUO CCD diffractometer	8397 reflections with *I* > 2σ(*I*)
Radiation source: fine-focus sealed tube	*R*_int_ = 0.026
phi and ω scans	θ_max_ = 27.5°, θ_min_ = 1.7°
Absorption correction: analytical based on measured indexed crystal faces (SHELXTL2014; Sheldrick, 2015*a*)	*h* = −30→30
*T*_min_ = 0.898, *T*_max_ = 0.947	*k* = −9→9
26031 measured reflections	*l* = −27→29
9018 independent reflections	

### Refinement

**Table:**

Refinement on *F*^2^	Secondary atom site location: difference Fourier map
Least-squares matrix: full	Hydrogen site location: mixed
*R*[*F*^2^ > 2σ(*F*^2^)] = 0.028	H atoms treated by a mixture of independent and constrained refinement
*wR*(*F*^2^) = 0.070	*w* = 1/[σ^2^(*F*_o_^2^) + (0.0456*P*)^2^] where *P* = (*F*_o_^2^ + 2*F*_c_^2^)/3
*S* = 1.02	(Δ/σ)_max_ = 0.001
9018 reflections	Δρ_max_ = 1.10 e Å^−^^3^
519 parameters	Δρ_min_ = −0.22 e Å^−^^3^
2 restraints	Absolute structure: Flack *x* determined using 3859 quotients [(*I*^+^)-(*I*^-^)]/[(*I*^+^)+(*I*^-^)] (Parsons *et al.*, 2013)
Primary atom site location: structure-invariant direct methods	Absolute structure parameter: 0.016 (4)

### Special details

**Table:**

Geometry. All esds (except the esd in the dihedral angle between two l.s. planes) are estimated using the full covariance matrix. The cell esds are taken into account individually in the estimation of esds in distances, angles and torsion angles; correlations between esds in cell parameters are only used when they are defined by crystal symmetry. An approximate (isotropic) treatment of cell esds is used for estimating esds involving l.s. planes.
Refinement. All H atoms were positioned geometrically ( C—H = 0.93/1.00 Å) and allowed to ride with *U*_iso_(H)= 1.2/1.5*U*_eq_(C). Methyl ones were allowed to rotate around the corresponding C—C. The asymmetric unit consists of a 2 Co units with each coordinated to four diamine ligand forming a square plane and two water ligands trans to each other.. The asymmetric unit also contains two tetraethylammonium counterions. Two nitrogen atoms, N3/N3' and N55/N56, were disordered and refined in two parts each with their site occupation factors dependently refined. All of the water protons were obtained from a Difference Fourier map and refined freely.

### Fractional atomic coordinates and isotropic or equivalent isotropic displacement parameters (Å^2^)

**Table:**

	*x*	*y*	*z*	*U*_iso_*/*U*_eq_	Occ. (<1)
Co1	0.45296 (2)	1.02347 (5)	0.23954 (2)	0.01173 (10)	
Co2	0.70898 (2)	0.98381 (6)	0.49718 (2)	0.01198 (10)	
O1	0.42840 (11)	1.2865 (3)	0.21237 (11)	0.0182 (5)	
H1A	0.4119 (19)	1.345 (6)	0.235 (2)	0.038 (12)*	
H1B	0.4509 (17)	1.337 (6)	0.2020 (16)	0.020 (11)*	
O2	0.47620 (10)	0.7567 (3)	0.26745 (11)	0.0184 (5)	
H2A	0.4479 (15)	0.700 (5)	0.2772 (14)	0.014 (8)*	
H2B	0.4867 (16)	0.707 (6)	0.2440 (18)	0.020 (11)*	
O3	0.68557 (10)	0.7243 (3)	0.46262 (10)	0.0172 (4)	
H3A	0.6660 (17)	0.671 (5)	0.4820 (18)	0.022 (10)*	
H3B	0.717 (2)	0.673 (8)	0.462 (2)	0.066 (16)*	
O4	0.73049 (10)	1.2503 (3)	0.52824 (10)	0.0165 (4)	
H4A	0.7045 (17)	1.301 (6)	0.5321 (17)	0.024 (11)*	
H4B	0.748 (2)	1.305 (7)	0.507 (2)	0.041 (13)*	
N1	0.27918 (17)	0.9826 (4)	0.44658 (17)	0.0263 (8)	
N3	0.3496 (7)	1.110 (2)	0.3882 (6)	0.014 (3)*	0.33 (4)
N3'	0.3369 (4)	1.1453 (11)	0.3777 (3)	0.0170 (17)*	0.67 (4)
N5	0.38584 (12)	1.0199 (4)	0.29474 (13)	0.0172 (6)	
N6	0.50206 (11)	1.1338 (3)	0.31233 (11)	0.0186 (5)	
N8	0.55339 (11)	1.2440 (4)	0.40377 (11)	0.0198 (6)	
N10	0.63870 (10)	1.1001 (3)	0.45012 (11)	0.0176 (5)	
N11	0.77978 (10)	0.8675 (3)	0.54269 (11)	0.0185 (5)	
N13	0.87084 (11)	0.7274 (4)	0.57243 (12)	0.0275 (7)	
N15	0.90600 (10)	0.5935 (3)	0.66723 (11)	0.0179 (5)	
N16	0.66067 (11)	0.9433 (4)	0.56980 (12)	0.0165 (5)	
N18	0.61244 (11)	0.8416 (4)	0.65572 (12)	0.0208 (6)	
N20	0.52420 (11)	0.9548 (4)	0.69062 (12)	0.0171 (5)	
N21	0.75691 (12)	1.0268 (4)	0.42431 (13)	0.0172 (6)	
N23	0.79811 (11)	1.1485 (4)	0.33595 (11)	0.0219 (6)	
N25	0.88281 (14)	1.0353 (4)	0.29320 (16)	0.0243 (8)	
C2	0.30902 (13)	1.0444 (4)	0.41469 (13)	0.0167 (6)	
C4	0.36385 (12)	1.0633 (4)	0.33577 (13)	0.0158 (6)	
C7	0.52810 (12)	1.1808 (4)	0.35482 (13)	0.0144 (6)	
C9	0.59893 (12)	1.1634 (4)	0.42615 (12)	0.0143 (6)	
C12	0.82135 (13)	0.7985 (4)	0.55973 (13)	0.0171 (6)	
C14	0.88662 (12)	0.6596 (4)	0.62471 (13)	0.0175 (6)	
C17	0.63568 (12)	0.9025 (4)	0.60908 (13)	0.0138 (6)	
C19	0.56523 (12)	0.9077 (4)	0.67179 (13)	0.0151 (6)	
C22	0.77826 (12)	1.0776 (4)	0.38380 (13)	0.0151 (6)	
C24	0.84342 (13)	1.0826 (4)	0.31572 (13)	0.0175 (6)	
N51	0.63136 (15)	0.4857 (4)	0.53742 (16)	0.0268 (8)	
C52	0.60294 (12)	0.4231 (4)	0.57142 (14)	0.0159 (6)	
N53	0.57322 (12)	0.3337 (4)	0.60740 (13)	0.0256 (7)	
C54	0.54159 (14)	0.4164 (4)	0.64236 (16)	0.0223 (7)	
N55	0.5044 (5)	0.4734 (8)	0.6665 (5)	0.019 (2)*	0.48 (3)
N56	0.5199 (5)	0.4749 (9)	0.6832 (5)	0.021 (2)*	0.52 (3)
N100	0.20492 (11)	0.8554 (3)	0.74448 (12)	0.0167 (5)	
C101	0.21237 (15)	0.9688 (5)	0.80049 (17)	0.0272 (7)	
H10A	0.2457	1.0473	0.7982	0.033*	
H10B	0.2198	0.8854	0.8345	0.033*	
C102	0.16270 (18)	1.0892 (6)	0.8122 (2)	0.0451 (10)	
H10C	0.1710	1.1572	0.8491	0.068*	
H10D	0.1555	1.1751	0.7794	0.068*	
H10E	0.1296	1.0128	0.8158	0.068*	
C103	0.15273 (13)	0.7392 (5)	0.74406 (14)	0.0231 (7)	
H10F	0.1494	0.6673	0.7070	0.028*	
H10G	0.1199	0.8210	0.7436	0.028*	
C104	0.15073 (16)	0.6084 (5)	0.79603 (16)	0.0321 (8)	
H10H	0.1157	0.5392	0.7921	0.048*	
H10I	0.1824	0.5239	0.7964	0.048*	
H10J	0.1528	0.6779	0.8330	0.048*	
C105	0.19842 (15)	0.9746 (5)	0.68951 (17)	0.0283 (8)	
H10K	0.1927	0.8945	0.6544	0.034*	
H10L	0.1644	1.0500	0.6914	0.034*	
C106	0.24752 (17)	1.0999 (6)	0.6811 (2)	0.0452 (11)	
H10M	0.2399	1.1714	0.6449	0.068*	
H10N	0.2530	1.1825	0.7151	0.068*	
H10O	0.2814	1.0267	0.6780	0.068*	
C107	0.25726 (14)	0.7379 (4)	0.74309 (15)	0.0228 (7)	
H10P	0.2903	0.8193	0.7452	0.027*	
H10Q	0.2603	0.6602	0.7789	0.027*	
C108	0.25941 (16)	0.6164 (6)	0.68945 (17)	0.0352 (9)	
H10R	0.2944	0.5465	0.6925	0.053*	
H10S	0.2276	0.5321	0.6875	0.053*	
H10T	0.2577	0.6917	0.6536	0.053*	
N200	0.46116 (10)	0.7171 (3)	0.48654 (11)	0.0159 (5)	
C201	0.47345 (14)	0.7741 (5)	0.42451 (14)	0.0251 (7)	
H20A	0.4679	0.9076	0.4206	0.030*	
H20B	0.4461	0.7140	0.3958	0.030*	
C202	0.53259 (15)	0.7274 (5)	0.40827 (15)	0.0274 (7)	
H20C	0.5373	0.7682	0.3678	0.041*	
H20D	0.5600	0.7890	0.4357	0.041*	
H20E	0.5383	0.5951	0.4109	0.041*	
C203	0.50310 (13)	0.7976 (4)	0.53272 (15)	0.0226 (7)	
H20F	0.5410	0.7536	0.5253	0.027*	
H20G	0.4945	0.7531	0.5722	0.027*	
C204	0.50327 (14)	1.0055 (4)	0.53314 (15)	0.0235 (7)	
H20H	0.5311	1.0493	0.5638	0.035*	
H20I	0.5126	1.0507	0.4945	0.035*	
H20J	0.4662	1.0502	0.5414	0.035*	
C205	0.40225 (13)	0.7789 (5)	0.49619 (16)	0.0274 (7)	
H20K	0.3762	0.7217	0.4656	0.033*	
H20L	0.4000	0.9127	0.4904	0.033*	
C206	0.38269 (14)	0.7340 (5)	0.55649 (15)	0.0269 (7)	
H20M	0.3444	0.7787	0.5588	0.040*	
H20N	0.3836	0.6015	0.5624	0.040*	
H20O	0.4075	0.7929	0.5872	0.040*	
C207	0.46624 (15)	0.5084 (4)	0.49254 (15)	0.0232 (7)	
H20P	0.5051	0.4724	0.4861	0.028*	
H20Q	0.4591	0.4736	0.5335	0.028*	
C208	0.42651 (15)	0.4023 (5)	0.44990 (16)	0.0281 (7)	
H20R	0.4322	0.2712	0.4564	0.042*	
H20S	0.3878	0.4343	0.4566	0.042*	
H20T	0.4338	0.4331	0.4092	0.042*	

### Atomic displacement parameters (Å^2^)

**Table:**

	*U*^11^	*U*^22^	*U*^33^	*U*^12^	*U*^13^	*U*^23^
Co1	0.0100 (2)	0.0133 (2)	0.0120 (2)	−0.00160 (18)	0.00139 (15)	−0.00165 (18)
Co2	0.0105 (2)	0.0140 (2)	0.0115 (2)	0.00317 (17)	0.00170 (15)	0.00155 (17)
O1	0.0210 (12)	0.0161 (11)	0.0181 (12)	0.0003 (9)	0.0053 (10)	0.0000 (9)
O2	0.0159 (11)	0.0179 (11)	0.0220 (13)	−0.0016 (9)	0.0047 (9)	−0.0020 (10)
O3	0.0160 (11)	0.0181 (11)	0.0179 (11)	0.0010 (9)	0.0040 (9)	0.0015 (9)
O4	0.0160 (11)	0.0166 (11)	0.0172 (11)	0.0028 (9)	0.0040 (9)	0.0019 (9)
N1	0.036 (2)	0.0171 (15)	0.0280 (18)	0.0040 (12)	0.0163 (15)	−0.0004 (12)
N5	0.0148 (13)	0.0189 (13)	0.0182 (15)	0.0002 (10)	0.0040 (11)	0.0019 (10)
N6	0.0166 (12)	0.0209 (13)	0.0181 (13)	0.0018 (10)	0.0000 (10)	−0.0024 (10)
N8	0.0200 (13)	0.0219 (13)	0.0165 (13)	0.0101 (10)	−0.0055 (10)	−0.0058 (10)
N10	0.0167 (12)	0.0200 (13)	0.0156 (13)	0.0033 (10)	−0.0011 (10)	−0.0006 (10)
N11	0.0150 (12)	0.0217 (13)	0.0187 (13)	0.0045 (10)	0.0002 (10)	0.0035 (10)
N13	0.0185 (13)	0.0472 (18)	0.0166 (13)	0.0160 (12)	0.0014 (10)	0.0109 (13)
N15	0.0158 (12)	0.0201 (12)	0.0176 (13)	0.0034 (10)	−0.0003 (10)	0.0025 (10)
N16	0.0149 (12)	0.0180 (13)	0.0168 (13)	0.0032 (10)	0.0026 (10)	0.0012 (10)
N18	0.0173 (13)	0.0262 (14)	0.0199 (13)	0.0081 (10)	0.0088 (10)	0.0083 (11)
N20	0.0164 (13)	0.0173 (13)	0.0182 (14)	0.0015 (10)	0.0047 (10)	0.0020 (10)
N21	0.0166 (14)	0.0193 (13)	0.0161 (14)	0.0031 (11)	0.0041 (11)	0.0010 (11)
N23	0.0245 (14)	0.0232 (13)	0.0196 (13)	0.0058 (11)	0.0118 (11)	0.0056 (11)
N25	0.0240 (18)	0.0209 (15)	0.0301 (19)	−0.0002 (12)	0.0163 (14)	0.0009 (13)
C2	0.0163 (14)	0.0173 (15)	0.0164 (15)	0.0016 (11)	0.0006 (12)	−0.0048 (12)
C4	0.0113 (13)	0.0183 (15)	0.0177 (16)	−0.0053 (11)	−0.0009 (12)	0.0007 (11)
C7	0.0128 (13)	0.0138 (13)	0.0170 (15)	0.0038 (10)	0.0044 (11)	0.0009 (11)
C9	0.0170 (14)	0.0146 (13)	0.0116 (13)	−0.0007 (11)	0.0026 (11)	−0.0033 (10)
C12	0.0188 (14)	0.0199 (14)	0.0129 (14)	0.0018 (12)	0.0029 (11)	0.0019 (11)
C14	0.0118 (13)	0.0198 (14)	0.0209 (16)	0.0037 (11)	0.0020 (11)	−0.0002 (12)
C17	0.0122 (13)	0.0129 (13)	0.0161 (14)	0.0035 (11)	−0.0012 (11)	−0.0015 (11)
C19	0.0181 (14)	0.0135 (13)	0.0136 (14)	−0.0020 (11)	0.0009 (11)	0.0019 (11)
C22	0.0129 (13)	0.0147 (14)	0.0175 (15)	0.0010 (11)	−0.0006 (11)	−0.0043 (11)
C24	0.0238 (16)	0.0141 (14)	0.0148 (14)	−0.0062 (12)	0.0030 (12)	0.0008 (11)
N51	0.0297 (18)	0.0194 (15)	0.033 (2)	0.0054 (12)	0.0170 (15)	0.0012 (12)
C52	0.0133 (14)	0.0149 (14)	0.0193 (16)	0.0033 (11)	0.0000 (12)	−0.0024 (12)
N53	0.0308 (16)	0.0194 (12)	0.0284 (17)	−0.0060 (12)	0.0141 (13)	−0.0038 (11)
C54	0.0228 (17)	0.0163 (15)	0.0286 (18)	−0.0019 (13)	0.0079 (14)	0.0054 (14)
N100	0.0173 (12)	0.0121 (11)	0.0197 (12)	−0.0023 (10)	−0.0051 (9)	0.0019 (10)
C101	0.0271 (18)	0.0238 (17)	0.0300 (19)	−0.0022 (14)	−0.0017 (14)	−0.0083 (14)
C102	0.040 (2)	0.037 (2)	0.058 (3)	0.0070 (18)	0.0062 (19)	−0.019 (2)
C103	0.0198 (15)	0.0241 (16)	0.0243 (17)	−0.0068 (13)	−0.0047 (12)	0.0014 (13)
C104	0.036 (2)	0.0275 (18)	0.032 (2)	−0.0112 (15)	−0.0030 (15)	0.0106 (15)
C105	0.0258 (17)	0.0258 (17)	0.032 (2)	0.0015 (14)	−0.0039 (14)	0.0150 (14)
C106	0.032 (2)	0.043 (2)	0.061 (3)	−0.0080 (18)	−0.0004 (19)	0.032 (2)
C107	0.0222 (16)	0.0209 (16)	0.0240 (17)	0.0016 (13)	−0.0073 (13)	0.0006 (13)
C108	0.0326 (19)	0.039 (2)	0.033 (2)	0.0088 (16)	−0.0046 (16)	−0.0140 (17)
N200	0.0141 (12)	0.0192 (13)	0.0138 (12)	0.0007 (10)	−0.0027 (9)	0.0002 (10)
C201	0.0300 (17)	0.0254 (16)	0.0189 (16)	−0.0012 (14)	−0.0043 (13)	0.0007 (13)
C202	0.0338 (19)	0.0223 (16)	0.0275 (18)	0.0004 (14)	0.0110 (14)	0.0011 (14)
C203	0.0188 (15)	0.0259 (16)	0.0217 (16)	0.0006 (12)	−0.0073 (12)	0.0003 (13)
C204	0.0212 (16)	0.0268 (17)	0.0224 (17)	−0.0044 (13)	0.0004 (13)	−0.0068 (13)
C205	0.0183 (16)	0.0301 (17)	0.033 (2)	0.0053 (13)	−0.0045 (14)	−0.0056 (15)
C206	0.0223 (16)	0.0280 (17)	0.0312 (18)	−0.0002 (13)	0.0074 (13)	−0.0048 (15)
C207	0.0259 (17)	0.0192 (16)	0.0239 (17)	0.0022 (13)	−0.0015 (14)	0.0019 (12)
C208	0.0305 (18)	0.0243 (17)	0.0290 (18)	−0.0061 (14)	−0.0002 (14)	−0.0082 (15)

### Geometric parameters (Å, º)

**Table:**

Co1—O1	2.094 (2)	C101—C102	1.522 (5)
Co1—N15^i^	2.099 (2)	C101—H10A	0.9900
Co1—N6	2.113 (2)	C101—H10B	0.9900
Co1—N20^ii^	2.114 (3)	C102—H10C	0.9800
Co1—N5	2.114 (3)	C102—H10D	0.9800
Co1—O2	2.116 (2)	C102—H10E	0.9800
Co2—N11	2.099 (2)	C103—C104	1.523 (5)
Co2—N10	2.105 (2)	C103—H10F	0.9900
Co2—N16	2.107 (3)	C103—H10G	0.9900
Co2—N21	2.109 (3)	C104—H10H	0.9800
Co2—O3	2.116 (2)	C104—H10I	0.9800
Co2—O4	2.126 (2)	C104—H10J	0.9800
O1—H1A	0.79 (5)	C105—C106	1.517 (5)
O1—H1B	0.71 (4)	C105—H10K	0.9900
O2—H2A	0.84 (4)	C105—H10L	0.9900
O2—H2B	0.71 (4)	C106—H10M	0.9800
O3—H3A	0.77 (4)	C106—H10N	0.9800
O3—H3B	0.84 (6)	C106—H10O	0.9800
O4—H4A	0.74 (4)	C107—C108	1.512 (5)
O4—H4B	0.77 (5)	C107—H10P	0.9900
N1—C2	1.149 (5)	C107—H10Q	0.9900
N3—C2	1.278 (9)	C108—H10R	0.9800
N3—C4	1.307 (9)	C108—H10S	0.9800
N3'—C4	1.334 (6)	C108—H10T	0.9800
N3'—C2	1.335 (6)	N200—C203	1.515 (4)
N5—C4	1.149 (4)	N200—C205	1.516 (4)
N6—C7	1.160 (4)	N200—C201	1.518 (4)
N8—C7	1.308 (4)	N200—C207	1.539 (4)
N8—C9	1.309 (4)	C201—C202	1.532 (5)
N10—C9	1.158 (4)	C201—H20A	0.9900
N11—C12	1.157 (4)	C201—H20B	0.9900
N13—C12	1.308 (4)	C202—H20C	0.9800
N13—C14	1.314 (4)	C202—H20D	0.9800
N15—C14	1.145 (4)	C202—H20E	0.9800
N15—Co1^iii^	2.099 (2)	C203—C204	1.524 (4)
N16—C17	1.151 (4)	C203—H20F	0.9900
N18—C19	1.309 (4)	C203—H20G	0.9900
N18—C17	1.312 (4)	C204—H20H	0.9800
N20—C19	1.155 (4)	C204—H20I	0.9800
N20—Co1^iv^	2.114 (3)	C204—H20J	0.9800
N21—C22	1.148 (4)	C205—C206	1.516 (5)
N23—C24	1.304 (4)	C205—H20K	0.9900
N23—C22	1.324 (4)	C205—H20L	0.9900
N25—C24	1.161 (5)	C206—H20M	0.9800
N51—C52	1.162 (5)	C206—H20N	0.9800
C52—N53	1.301 (4)	C206—H20O	0.9800
N53—C54	1.290 (4)	C207—C208	1.518 (5)
C54—N55	1.159 (8)	C207—H20P	0.9900
C54—N56	1.176 (9)	C207—H20Q	0.9900
N100—C103	1.513 (4)	C208—H20R	0.9800
N100—C101	1.517 (4)	C208—H20S	0.9800
N100—C105	1.520 (4)	C208—H20T	0.9800
N100—C107	1.524 (4)		
			
O1—Co1—N15^i^	91.39 (10)	N100—C103—C104	115.1 (3)
O1—Co1—N6	90.28 (10)	N100—C103—H10F	108.5
N15^i^—Co1—N6	178.10 (10)	C104—C103—H10F	108.5
O1—Co1—N20^ii^	89.81 (11)	N100—C103—H10G	108.5
N15^i^—Co1—N20^ii^	91.57 (10)	C104—C103—H10G	108.5
N6—Co1—N20^ii^	87.54 (10)	H10F—C103—H10G	107.5
O1—Co1—N5	88.62 (11)	C103—C104—H10H	109.5
N15^i^—Co1—N5	94.04 (10)	C103—C104—H10I	109.5
N6—Co1—N5	86.90 (11)	H10H—C104—H10I	109.5
N20^ii^—Co1—N5	174.22 (11)	C103—C104—H10J	109.5
O1—Co1—O2	178.90 (11)	H10H—C104—H10J	109.5
N15^i^—Co1—O2	88.25 (10)	H10I—C104—H10J	109.5
N6—Co1—O2	90.09 (10)	C106—C105—N100	114.8 (3)
N20^ii^—Co1—O2	91.25 (10)	C106—C105—H10K	108.6
N5—Co1—O2	90.36 (10)	N100—C105—H10K	108.6
N11—Co2—N10	178.97 (11)	C106—C105—H10L	108.6
N11—Co2—N16	91.79 (10)	N100—C105—H10L	108.6
N10—Co2—N16	89.21 (10)	H10K—C105—H10L	107.6
N11—Co2—N21	88.68 (11)	C105—C106—H10M	109.5
N10—Co2—N21	90.32 (11)	C105—C106—H10N	109.5
N16—Co2—N21	179.42 (12)	H10M—C106—H10N	109.5
N11—Co2—O3	89.94 (9)	C105—C106—H10O	109.5
N10—Co2—O3	89.81 (10)	H10M—C106—H10O	109.5
N16—Co2—O3	90.95 (10)	H10N—C106—H10O	109.5
N21—Co2—O3	89.38 (10)	C108—C107—N100	115.4 (3)
N11—Co2—O4	92.49 (10)	C108—C107—H10P	108.4
N10—Co2—O4	87.74 (9)	N100—C107—H10P	108.4
N16—Co2—O4	90.26 (10)	C108—C107—H10Q	108.4
N21—Co2—O4	89.39 (10)	N100—C107—H10Q	108.4
O3—Co2—O4	177.25 (10)	H10P—C107—H10Q	107.5
Co1—O1—H1A	117 (3)	C107—C108—H10R	109.5
Co1—O1—H1B	112 (3)	C107—C108—H10S	109.5
H1A—O1—H1B	111 (5)	H10R—C108—H10S	109.5
Co1—O2—H2A	110 (2)	C107—C108—H10T	109.5
Co1—O2—H2B	110 (3)	H10R—C108—H10T	109.5
H2A—O2—H2B	107 (4)	H10S—C108—H10T	109.5
Co2—O3—H3A	113 (3)	C203—N200—C205	111.1 (2)
Co2—O3—H3B	101 (4)	C203—N200—C201	111.6 (2)
H3A—O3—H3B	112 (5)	C205—N200—C201	107.5 (2)
Co2—O4—H4A	108 (3)	C203—N200—C207	106.4 (2)
Co2—O4—H4B	113 (3)	C205—N200—C207	110.6 (3)
H4A—O4—H4B	108 (5)	C201—N200—C207	109.6 (2)
C2—N3—C4	126.2 (7)	N200—C201—C202	114.3 (3)
C4—N3'—C2	119.5 (6)	N200—C201—H20A	108.7
C4—N5—Co1	153.4 (2)	C202—C201—H20A	108.7
C7—N6—Co1	174.2 (2)	N200—C201—H20B	108.7
C7—N8—C9	119.2 (2)	C202—C201—H20B	108.7
C9—N10—Co2	177.4 (2)	H20A—C201—H20B	107.6
C12—N11—Co2	170.1 (2)	C201—C202—H20C	109.5
C12—N13—C14	122.9 (3)	C201—C202—H20D	109.5
C14—N15—Co1^iii^	171.5 (2)	H20C—C202—H20D	109.5
C17—N16—Co2	173.0 (2)	C201—C202—H20E	109.5
C19—N18—C17	122.0 (3)	H20C—C202—H20E	109.5
C19—N20—Co1^iv^	164.3 (2)	H20D—C202—H20E	109.5
C22—N21—Co2	168.5 (3)	N200—C203—C204	113.3 (3)
C24—N23—C22	120.3 (3)	N200—C203—H20F	108.9
N1—C2—N3	168.3 (10)	C204—C203—H20F	108.9
N1—C2—N3'	168.5 (5)	N200—C203—H20G	108.9
N5—C4—N3	167.7 (9)	C204—C203—H20G	108.9
N5—C4—N3'	168.6 (5)	H20F—C203—H20G	107.7
N6—C7—N8	174.4 (3)	C203—C204—H20H	109.5
N10—C9—N8	174.4 (3)	C203—C204—H20I	109.5
N11—C12—N13	172.4 (3)	H20H—C204—H20I	109.5
N15—C14—N13	171.8 (3)	C203—C204—H20J	109.5
N16—C17—N18	172.7 (3)	H20H—C204—H20J	109.5
N20—C19—N18	173.3 (3)	H20I—C204—H20J	109.5
N21—C22—N23	173.7 (3)	N200—C205—C206	115.0 (3)
N25—C24—N23	173.5 (4)	N200—C205—H20K	108.5
N51—C52—N53	173.0 (3)	C206—C205—H20K	108.5
C54—N53—C52	121.7 (3)	N200—C205—H20L	108.5
N55—C54—N53	165.8 (7)	C206—C205—H20L	108.5
N56—C54—N53	166.1 (8)	H20K—C205—H20L	107.5
C103—N100—C101	110.9 (3)	C205—C206—H20M	109.5
C103—N100—C105	106.6 (2)	C205—C206—H20N	109.5
C101—N100—C105	111.7 (2)	H20M—C206—H20N	109.5
C103—N100—C107	111.4 (2)	C205—C206—H20O	109.5
C101—N100—C107	106.4 (2)	H20M—C206—H20O	109.5
C105—N100—C107	109.9 (3)	H20N—C206—H20O	109.5
N100—C101—C102	114.9 (3)	C208—C207—N200	114.2 (3)
N100—C101—H10A	108.5	C208—C207—H20P	108.7
C102—C101—H10A	108.5	N200—C207—H20P	108.7
N100—C101—H10B	108.5	C208—C207—H20Q	108.7
C102—C101—H10B	108.5	N200—C207—H20Q	108.7
H10A—C101—H10B	107.5	H20P—C207—H20Q	107.6
C101—C102—H10C	109.5	C207—C208—H20R	109.5
C101—C102—H10D	109.5	C207—C208—H20S	109.5
H10C—C102—H10D	109.5	H20R—C208—H20S	109.5
C101—C102—H10E	109.5	C207—C208—H20T	109.5
H10C—C102—H10E	109.5	H20R—C208—H20T	109.5
H10D—C102—H10E	109.5	H20S—C208—H20T	109.5
			
C4—N3—C2—N1	132 (2)	C107—N100—C105—C106	−58.1 (4)
C4—N3'—C2—N1	−148.8 (18)	C103—N100—C107—C108	60.6 (4)
Co1—N5—C4—N3	−39 (3)	C101—N100—C107—C108	−178.4 (3)
Co1—N5—C4—N3'	73 (2)	C105—N100—C107—C108	−57.3 (4)
C2—N3—C4—N5	−131.3 (19)	C203—N200—C201—C202	55.9 (3)
C2—N3'—C4—N5	154.7 (17)	C205—N200—C201—C202	178.0 (3)
C52—N53—C54—N55	114 (2)	C207—N200—C201—C202	−61.7 (3)
C52—N53—C54—N56	−118 (2)	C205—N200—C203—C204	−59.1 (4)
C103—N100—C101—C102	−55.4 (4)	C201—N200—C203—C204	60.9 (3)
C105—N100—C101—C102	63.4 (4)	C207—N200—C203—C204	−179.6 (3)
C107—N100—C101—C102	−176.7 (3)	C203—N200—C205—C206	−57.3 (4)
C101—N100—C103—C104	−58.1 (4)	C201—N200—C205—C206	−179.7 (3)
C105—N100—C103—C104	−180.0 (3)	C207—N200—C205—C206	60.6 (4)
C107—N100—C103—C104	60.2 (4)	C203—N200—C207—C208	179.3 (3)
C103—N100—C105—C106	−178.8 (3)	C205—N200—C207—C208	58.5 (4)
C101—N100—C105—C106	59.8 (4)	C201—N200—C207—C208	−59.9 (4)

Symmetry codes: (i) *x*−1/2, −*y*+3/2, *z*−1/2; (ii) *x*, −*y*+2, *z*−1/2; (iii) *x*+1/2, −*y*+3/2, *z*+1/2; (iv) *x*, −*y*+2, *z*+1/2.

### Hydrogen-bond geometry (Å, º)

**Table:**

*D*—H···*A*	*D*—H	H···*A*	*D*···*A*	*D*—H···*A*
O1—H1*A*···N25^v^	0.79 (5)	2.08 (5)	2.862 (4)	168 (4)
O1—H1*B*···N55^ii^	0.71 (4)	2.09 (4)	2.792 (7)	168 (4)
O1—H1*B*···N56^ii^	0.71 (4)	2.22 (4)	2.922 (9)	170 (4)
O2—H2*A*···N25^vi^	0.84 (4)	2.03 (4)	2.862 (4)	172 (3)
O2—H2*B*···N55^vii^	0.71 (4)	2.27 (4)	2.963 (8)	169 (4)
O2—H2*B*···N56^vii^	0.71 (4)	2.12 (4)	2.819 (8)	171 (4)
O3—H3*A*···N51	0.77 (4)	2.07 (4)	2.822 (4)	165 (4)
O3—H3*B*···N1^viii^	0.84 (6)	2.09 (6)	2.904 (4)	162 (5)
O4—H4*A*···N51^ix^	0.74 (4)	2.23 (4)	2.958 (4)	172 (4)
O4—H4*B*···N1^x^	0.77 (5)	2.07 (5)	2.833 (4)	169 (5)
C105—H10*L*···N18^v^	0.99	2.57	3.439 (5)	146
C202—H20*E*···N8^xi^	0.98	2.60	3.579 (5)	173
C204—H20*H*···N53^ix^	0.98	2.49	3.316 (4)	142

Symmetry codes: (ii) *x*, −*y*+2, *z*−1/2; (v) *x*−1/2, *y*+1/2, *z*; (vi) *x*−1/2, *y*−1/2, *z*; (vii) *x*, −*y*+1, *z*−1/2; (viii) *x*+1/2, *y*−1/2, *z*; (ix) *x*, *y*+1, *z*; (x) *x*+1/2, *y*+1/2, *z*; (xi) *x*, *y*−1, *z*.
